# GENDER DISPARITIES IN VISUAL DIFFICULTY AMONG OLDER ADULTS: A CROSS-NATIONAL ANALYSIS

**DOI:** 10.1093/geroni/igae098.2151

**Published:** 2024-12-31

**Authors:** Shane Burns, Joseph Saenz, Joshua Ehrlich

**Affiliations:** University of Michigan, Ann Arbor, Michigan, United States; Arizona State University, Phoenix, Arizona, United States; University of Michigan, Ann Arbor, Michigan, United States

## Abstract

**Background:**

Rapid global aging will lead to an increase of older adults with visual difficulty, especially women. However, little is known what contextual factors drive this gendered distinction across the world.

**Methods:**

Using data from the Health & Retirement Study (HRS) international family of studies, we investigate gender disparities in visual difficulty among respondents ages 55-89 in the United States (n=14,452), England (n=5,708), Mexico (n=10,972), and Korea (n=6,649) while adjusting for demographic, socioeconomic, and health characteristics.

**Results:**

Reports of visual difficulty across age groups were generally highest in Mexico, followed by Korea, the United States, and England. In the United States, women had a visual difficulty disadvantage that was fully explained by education and socioeconomic indicators. In England, women had a visual difficulty disadvantage that was partially explained by education and socioeconomic indicators. In Mexico, men had a visual difficulty disadvantage that was explained by comorbidities. In Korea, women had a visual difficulty disadvantage that was explained by comorbidities and health behaviors. Conclusions: Our results highlight the heterogeneity of gender disparities in visual difficulty among older adults across the world.

